# CT Imaging Features of Acute Aortic Syndrome: A Case Series

**DOI:** 10.7759/cureus.103111

**Published:** 2026-02-06

**Authors:** Nidhi Patel, Ruhali Patel, Viral Patel, Achint Patel, Kushal Pujara

**Affiliations:** 1 Department of Radiodiagnosis, Pramukhswami Medical College and Shree Krishna Hospital, Bhaikaka University, Anand, IND; 2 Department of Radiodiagnosis, Smt. Kashibai Navale Medical College and General Hospital, Pune, IND; 3 Department of Cardiology, Pramukhswami Medical College and Shree Krishna Hospital, Bhaikaka University, Anand, IND

**Keywords:** acute aortic syndrome, aortic dissection, intramural hematoma, penetrating atherosclerotic ulcer, ruptured aortic dissection

## Abstract

Acute aortic syndrome (AAS) encompasses a spectrum of life-threatening aortic pathologies, including aortic dissection, intramural hematoma (IMH), and penetrating atherosclerotic ulcer (PAU). Due to overlapping and often nonspecific clinical presentations, timely and accurate imaging is essential for diagnosis and management. Multidetector computed tomography (MDCT) has emerged as the gold standard imaging modality for the evaluation of AAS, due to its rapid acquisition, high spatial resolution, and ability to assess the entire aorta and its branches in a single acquisition. This case series presents four distinct cases of AAS to highlight the critical role of MDCT in diagnosis and treatment planning. The first case is of aortic dissection (Stanford type A) extending into the iliac arteries with a thrombosed false lumen. The second case demonstrated an IMH (Stanford type B) with an ulcer-like projection and associated PAU. The third case shows a PAU (Stanford type B/DeBakey type III) in the infra-renal aorta, while the fourth involved a ruptured Stanford type B dissection with hemomediastinum and hemothorax. All patients underwent MDCT aortography with pre- and post-contrast phases, enabling detailed visualization of the aortic wall, lumen, branch vessels, and associated complications. Non-contrast imaging was pivotal in detecting acute hemorrhage, while contrast-enhanced phases allowed clear visualization of intimal flaps, true and false lumens, ulcerations, and involvement of branch vessels. This series highlights the indispensable role of MDCT in the early recognition and characterization of AAS, enabling prompt intervention and significantly reducing morbidity/mortality.

## Introduction

Acute aortic syndrome (AAS) includes interrelated emergent aortic conditions comprising aortic dissection, penetrating atherosclerotic ulcer (PAU), intramural hematoma (IMH), and unstable/ruptured aortic aneurysm. Timely and detailed evaluation of AAS is crucial in providing immediate medical or surgical intervention, which further aids in reducing morbidity and mortality[1]. The predictable incidence of AAS is 2.6-3.5 per 100,000 per year, with a male predominance and average age of presentation being 60 years [2].

Though excruciating chest pain of sudden onset is a classical presentation of AAS, symptoms alone are rarely diagnostic [3]. This is because the presentation can be highly variable depending on the vascular distribution affected. Involvement of the subclavian or iliac arteries may present with limb ischemia. On the other hand, involvement of the carotids may present with stroke-like symptoms. Involvement of the superior mesenteric artery or inferior mesenteric artery will cause mesenteric ischemia, presenting with severe abdominal pain responding poorly to analgesia. Thus, the AAS spectrum is often described as the "Great imitator." Syncope is a notable symptom of acute aortic dissection, often raising suspicion for complications such as cardiac tamponade, cerebral vessel involvement, or activation of cerebral baroreceptors [3]. Symptoms alone also do not allow differentiation between types of AAS and accompanying findings. Hence, multidetector computed tomography (MDCT) plays a critical role and is considered the gold standard first-line imaging modality in diagnosing, characterizing, and classifying AAS as well as guiding immediate management[4]. IMH and PAU can have an unpredictable course, thus requiring accurate diagnosis and a targeted therapeutic approach. Furthermore, MDCT aids in ruling out other differentials of acute chest pain, such as pneumothorax and pulmonary embolism.

In the present case series, we discuss four cases of AAS, namely, aortic dissection, IMH, PAU, and ruptured aortic dissection, and highlight the importance of MDCT in their timely diagnosis.

## Case presentation

Case 1: Aortic dissection with thrombus in the false lumen

**Figure 1:**
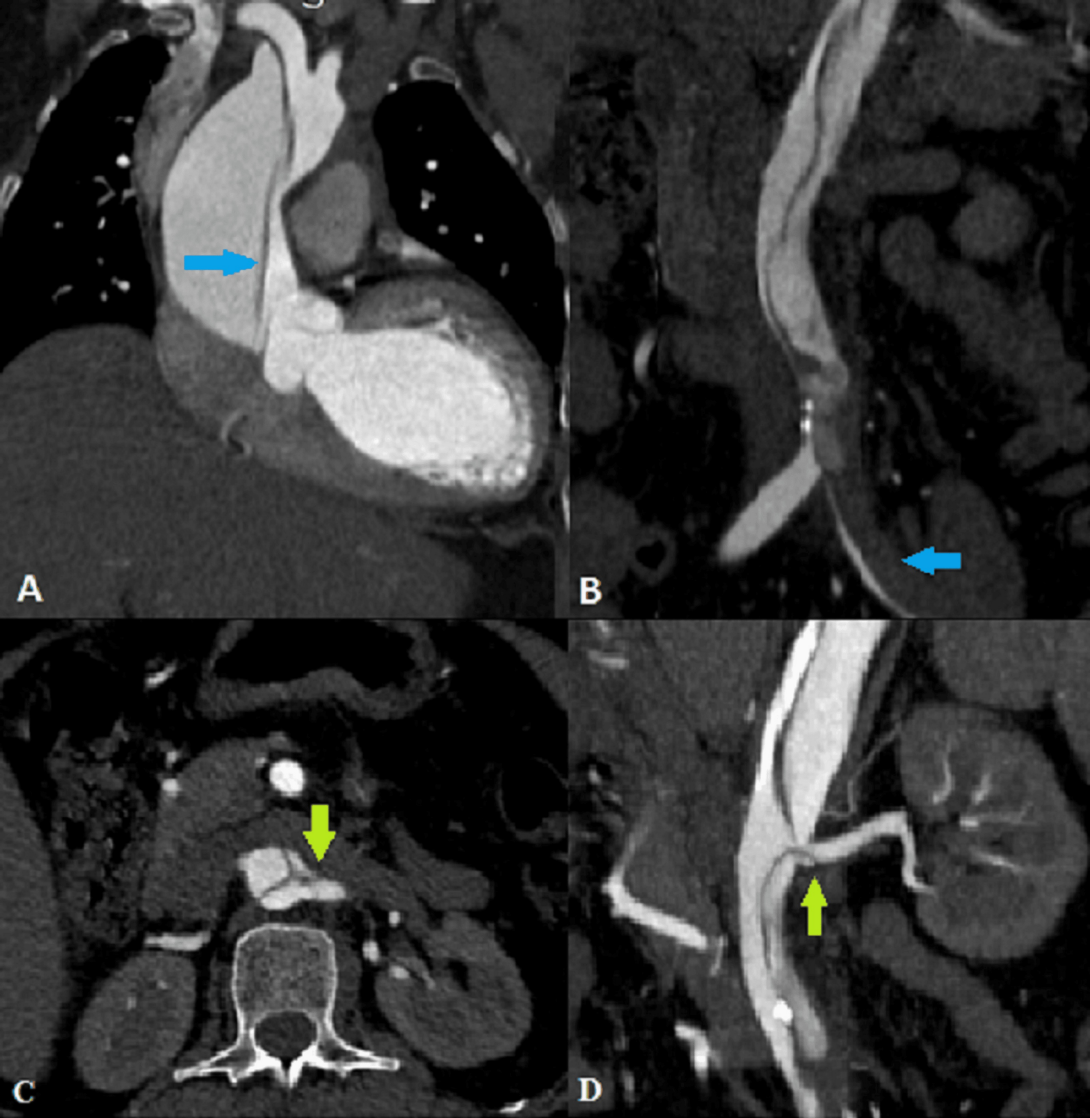
Coronal CT angiography images demonstrating (A) dissecting intimal flap in the ascending aorta (blue arrow) with differential enhancement of the true (smaller) and false (larger) lumens; (B) dissecting intimal flap extending into the abdominal aorta and left common iliac artery with thrombus in false lumen (blue arrow); (C) axial and (D) coronal CT angiography images demonstrating extension of intimal flap into the proximal left renal artery (green arrows).

A 61-year-old hypertensive female presented with abdominal pain and left lower limb weakness for one day. MDCT aortography (Figure 1) revealed a dissecting intimal flap involving the ascending aorta from the level of the sinotubular junction, arch of aorta, and descending aorta extending to the aortic bifurcation and into the left common iliac artery and proximal left external iliac artery (Stanford type A/DeBakey type 1) [5]. A non-enhancing thrombus was noted within the false lumen of the left common iliac artery, causing compression of the patent true lumen. The right brachiocephalic artery, left common carotid artery, left subclavian artery, superior mesenteric artery, inferior mesenteric artery, and right renal artery originated from the true lumen, while the coeliac trunk and left renal artery originated from the false lumen with extension of the intimal flap into the proximal portion of the left renal artery.

Case 2: Intramural hematoma

**Figure 2:**
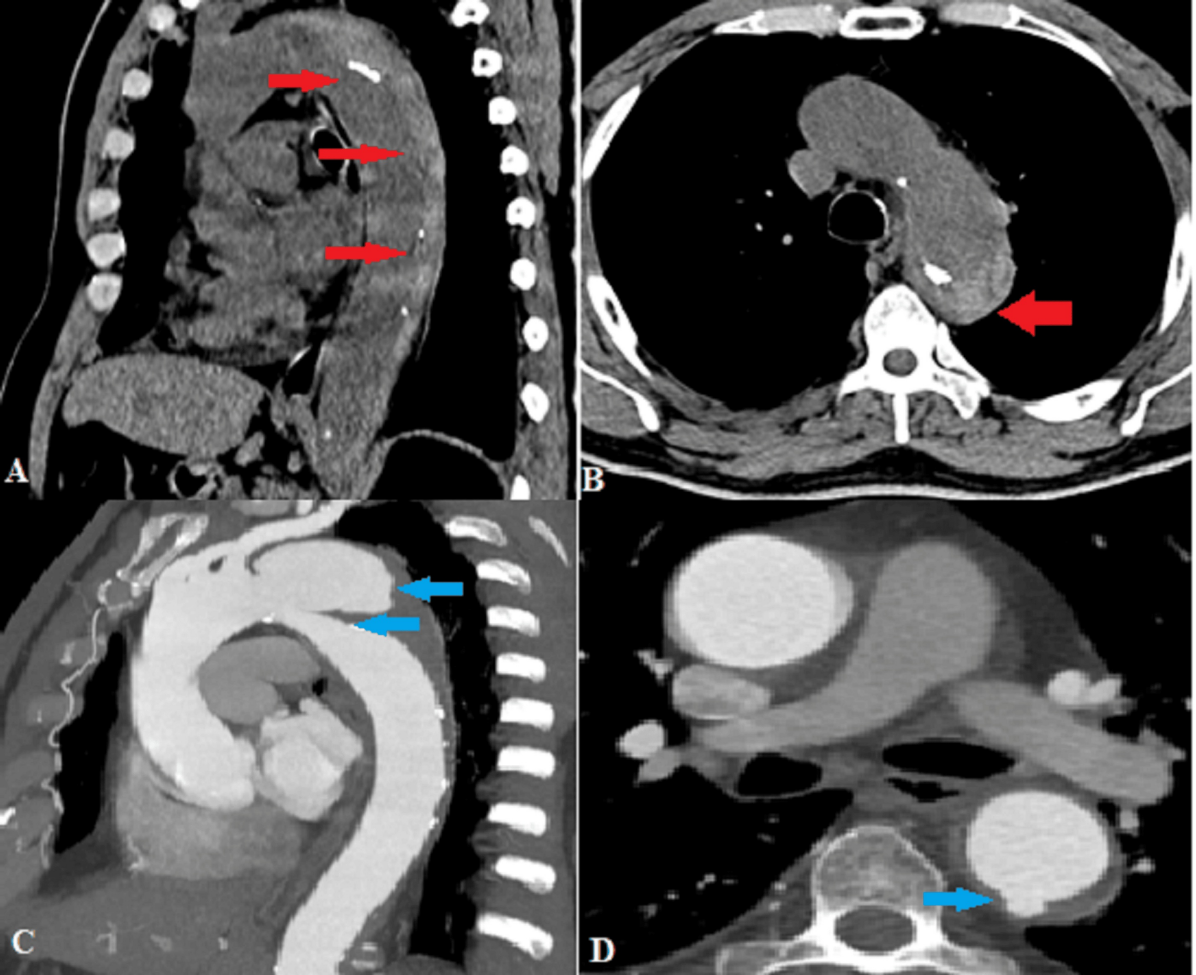
(A) Sagittal and (B) axial non-contrast CT images demonstrate crescentic hyperdensity along the posterior walls of the arch of the aorta and descending aorta (red arrows). Intimal calcifications surround the true lumen. (C) Sagittal CT angiography image shows an intramural hematoma complicated by an ulcer-like projection (blue arrows). (D) Axial CT angiography shows a contrast-filled ulcer crater in the posterior wall of the descending aorta (blue arrow).

A 67-year-old hypertensive, male smoker, presented with abrupt-onset chest pain radiating to the back. Non-contrast CT (Figures 2A, 2B) showed crescentic hyperdensity along the posterolateral wall of the arch of the aorta and descending thoracic aorta, measuring up to 20 mm in maximum thickness. It displaced the atherosclerotic intimal calcifications toward the aortic lumen. These features were consistent with intramural hematoma (Stanford type B)[5]. Further, MDCT aortography (Figures 2C, 2D) revealed a localized area of contrast enhancement extending from the aortic lumen into the IMH, with a visible communication (neck measuring 21 mm), just distal to the origin of the left subclavian artery, suggestive of an ulcer-like projection. In addition, a few small contrast-filled outpouchings were seen arising from the posterior wall of the descending thoracic aorta and left lateral wall of the abdominal aorta, consistent with penetrating atherosclerotic ulcers.

Case 3: Penetrating atherosclerotic ulcer

**Figure 3:**
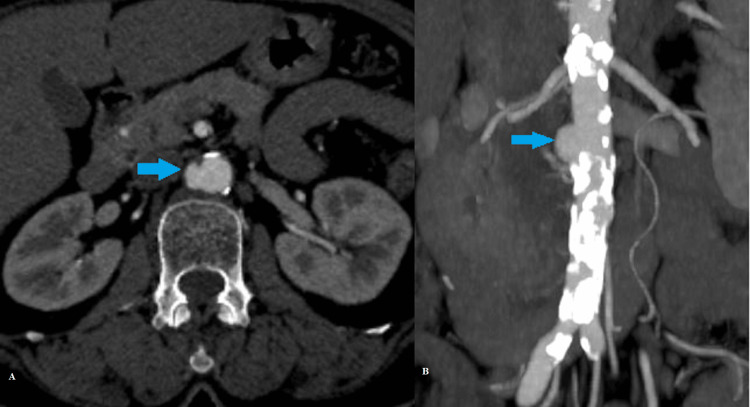
(A) Axial and (B) coronal CT angiography images show contrast-filled ulcer crater in the background of severe systemic atherosclerosis (blue arrows).

A 65-year-old hypertensive female presented with intense abdominal pain and shortness of breath. MDCT aortography (Figure 3) revealed a focal contrast-filled outpouching, representing a penetrating atherosclerotic ulcer along the right lateral wall of the infrarenal aorta in the background of extensive atherosclerotic wall calcifications. The ulcer measured 6.8 mm in depth and 12 mm in width (Stanford type B/DeBakey type III) [5].

Case 4: Ruptured aortic dissection

**Figure 4:**
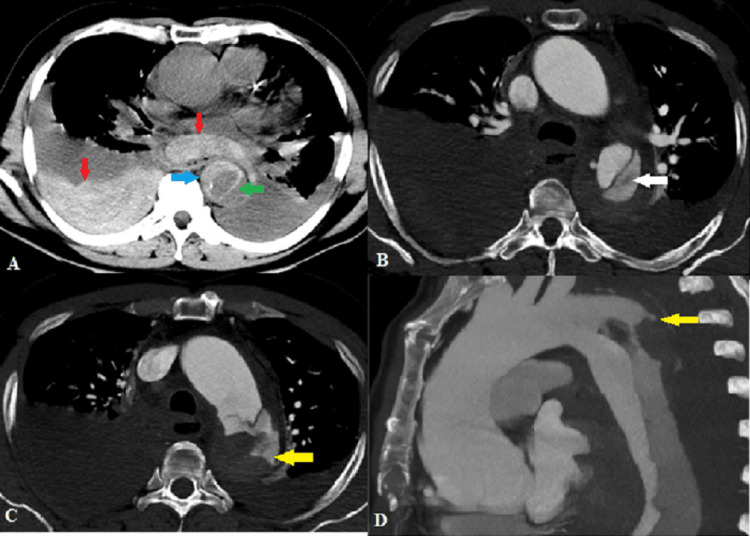
(A) Axial non-contrast CT shows a crescentic IMH (blue arrow) and dissecting intimal flap (green arrow) in the descending thoracic aorta with hemomediastinum and right hemothorax (red arrows). Axial CT angiography images show (B) dissecting intimal flap with faintly opacifying false lumen (white arrow) and (C) probable site of rupture along the left lateral wall of the aorta (yellow arrow). (D) Sagittal MIP image shows the site of rupture. IMH: intramural hematoma; MIP: maximum intensity projection.

A 54-year-old male presented with excruciating chest pain and breathlessness. Non-contrast CT (Figure 4A) showed a near-circumferential crescentic hyperdensity along the arch of the aorta and descending thoracic aorta, suggestive of intramural hematoma. A dissecting intimal flap was also seen in the arch of the aorta, descending thoracic aorta, and abdominal aorta. In addition, hemomediastinum and right hemothorax were noted, raising suspicion of a ruptured dissection. Further, MDCT aortography (Figures 4B-4D) revealed aortic dissection distal to the origin of the left subclavian artery (Stanford type B/DeBakey type III) [5]. The faintly enhancing false lumen in the arch of the aorta showed an irregular contour with a small focal rupture along the left lateral wall.

## Discussion

AAS comprises an array of disorders that affect the aortic wall and are precipitated by underlying abnormalities of the media or intima, trauma, or instrumentation [6]. Both acquired and genetic conditions share a common pathway leading to the breakdown in the integrity of the intima, weakening of the media layer, and increasing wall stress. The common acquired risk factors include hypertension, hyperlipidemia, diabetes, physical trauma, and smoking. Marfan’s syndrome, vascular Ehlers-Danlos syndrome, annuloaortic ectasia, bicuspid aortic valve, Turner syndrome, and familial aortic dissection are genetic conditions that predispose to premature degeneration of elastin and collagen within the vessel wall, making it more vulnerable to injury [4,7].

However, clinical presentation is highly variable and not always helpful in differentiating between the types of AAS. Clinically, it may also be confused with far more common acute conditions, such as myocardial infarction and pulmonary thromboembolism [6]. It is worth noting that many aortic dissections are missed in the emergency room; only 15% to 43% of verified cases are accurately diagnosed at first presentation [8]. Diagnostic imaging is thus crucial to confirm and precisely diagnose the type and extent of disease, and possible complications [6].

When AAS is suspected, a non-contrast CT from the thoracic inlet to slightly below the lesser trochanters is needed to visualize the entire aorta, proximal aortic arch branches, iliac arteries, and proximal femoral arteries. Post contrast imaging is then performed using a bolus triggering technique in the arterial phase (acquired at 15-25 seconds post contrast injection) and venous phase (acquired at 50-60 seconds post contrast injection) with the same pre-contrast anatomic coverage.

Wherever resources permit, ECG-gated imaging is recommended for the thoracic aorta in the arterial phase to minimize the cardiac and pulsation motion artefacts. Contrast injection is recommended at a faster rate of 3-5 mL/second. A saline chase is given after the contrast to limit contrast in the lumen of interest. To reduce streak artefacts from contrast in vessels crossing the arch, contrast injection is recommended in the right arm.

Non-contrast CT images that can be acquired in a span of a few seconds provide a window for the detection of acute hemorrhage. The hemorrhage may be present within the aortic wall, representing either IMH or a thrombosed false lumen. It can also be detected surrounding the aorta (periaortic hematoma), or in the mediastinum (hemomediastinum) or pleural space (hemothorax). Such findings are key pieces of diagnostic information that can be difficult to gather from contrast-enhanced images in the setting of AAS.

Contrast differences between the arterial and venous phases can aid in differentiating the true lumen from the false lumen.

It is crucial to visualize branches of the arch to evaluate the extent of dissection and risk of possible neurological and ischemic complications. The iliac vessels are included for evaluation of endovascular treatment possibilities.

Aortic dissection is characterized by an intimal tear, allowing blood to breach and disrupt the aortic media. A false lumen is subsequently formed parallel to the true aortic lumen, with the false lumen having pressure more than or equal to that of the true lumen. On MDCT, the diagnosis is based on the visualization of an intimal flap separating the true and false lumen. In addition to making the diagnosis, MDCT also assesses the extent of aortic, carotid, subclavian, visceral artery, and iliac artery involvement [3]. Identification of impending rupture is crucial as these patients are at increased risk for frank rupture and may benefit from further preoperative imaging, followed by urgent surgery [9]. Apart from ruptured dissection, the most common and detrimental complication is organ malperfusion, which can be predicted by demonstrating extension of the intimal flap in visceral branches.

The Stanford and DeBakey classification systems seek to both describe the pathology and guide clinical decision-making. The Stanford classification divides dissections into type A (involving the ascending aorta) and type B (involving only the descending aorta, distal to the left subclavian artery origin), with type A being a surgical emergency [5]. The DeBakey system is a more detailed, anatomical classification with type I involving both ascending and descending aorta, type II involving ascending aorta only, and type III involving only the descending aorta (commencing after the origin of the left subclavian artery) [5]. Types I and II warrant surgical management, while type III is generally managed medically with blood pressure control unless complicated by end-organ ischemia.

The European Association for Cardio-Thoracic Surgery and the Society of Thoracic Surgeons recommended the TEM (type-entry-malperfusion) classification as an extension of the Stanford classification in their 2024 guidelines. TEM classification incorporates the type (based on Stanford classification), entry tear location, and malperfusion status as its key parameters. It recognizes those dissections that arise in the aortic arch, but do not involve the ascending aorta as "Non-A-Non-B" type [10]. These make up approximately 10% of all aortic dissection cases [11]. Types A and B are described as per the Stanford classification. This system caters details required for the novel endovascular treatments by including the entry tear location. It also allows for better stratification of the urgency of treatment by including malperfusion status.

Management of acute aortic dissection depends largely on the extent of dissection, location of the primary entry tear, and whether or not there is evidence of organ malperfusion. ICU admission is crucial with placement of an arterial line in the unaffected limb for continuous blood pressure monitoring. Aggressive blood pressure control with the use of intravenous beta-blockers helps reduce shear stress on the aortic wall. In addition, analgesics like morphine are used for pain control and to reduce sympathetic tone.

Type A dissections are surgical emergencies owing to the risk of catastrophic complications such as cardiac tamponade, severe aortic regurgitation, myocardial infarction, or aortic rupture. Surgical intervention includes removing the primary tear site and obliterating the entry point into the false lumen to prevent further propagation. Aortic replacement using a synthetic graft with or without aortic valve repair/replacement may be required.

Since uncomplicated type B aortic dissection carries a relatively low risk of rupture or sudden death and surgical management is associated with high mortality and morbidity, medical treatment alone is advocated for uncomplicated type B dissection. Surgical intervention is reserved for patients with complicated type B dissections.

Thoracic endovascular aortic repair (TEVAR) is a newer, less invasive technique used for type A and complicated type B dissections in the setting of favorable aortic anatomy. Endovascular management of dissection comprises (1) aortic stent-graft placement, (2) dissection flap fenestration, and (3) branch-vessel stenting [12].

Stent-graft treatment aims at covering the primary intimal tear and creating a seal that stops blood flow into the false lumen. This will prevent the transmission of systemic pressure across the major intimal defect. If the seal is adequate, cardiac output is redirected into the true lumen, causing the false lumen to rapidly decompress [12].

Intimal flap fenestration is an alternative endovascular approach to stent-graft repair in aortic dissection, focusing on creating a distal re-entry rather than sealing the primary entry tear. By equalizing pressures between the true and false lumens, this technique will relieve the dynamic obstruction of the aorta and its branch vessels [12].

Branch-vessel stenting involves placing an uncovered stent via the aortic true lumen into the true lumen of the involved branch, with the stent covering the length of the dissected branch segment. On stent deployment, the flap is displaced, and a cylindrical true lumen is established [12].

Aortic IMH occurs due to a contained hemorrhage into the aortic wall, usually from the vasa vasorum, without a macroscopic or grossly visible intimal tear. Similar to the dissection of the aorta, aortic IMHs are also classified according to the Stanford classification, with type A associated with increased risk for complications, such as pericardial and/or pleural effusion, dissection, aneurysm formation, and death. On non-contrast CT, acute IMHs are seen as crescentic, hyperattenuating (60-70 HU) regions of eccentric aortic wall thickening, displacing the intimal calcifications inwards, toward the lumen. Management of IMH parallels that of classic aortic dissection, with surgical intervention recommended for type A IMH and medical therapy favored for type B IMH [12].

PAU is a deep atheromatous ulcer that penetrates through the elastic lamina and into the media. On CT aortography, it is seen as a focal contrast-filled out-pouching from the aortic wall or into the thickened aortic wall with no appreciable intimal flap or false lumen. The most common site for a PAU is the descending thoracic aorta, with signs of extensive atherosclerosis in the rest of the aorta and its major branches. These are again classified using the Stanford classification used for aortic dissections. PAUs with a depth >10 mm and width >20 mm are associated with a higher rate of progression to aortic dissection, aneurysm, and rupture. Although no universal treatment approach exists, early surgical graft replacement in the aorta has been advocated in symptomatic patients of PAU [12].

It is of paramount importance to differentiate PAU from ulcer-like projection (ULP), owing to the difference in prognosis and treatment planning. PAU is a deep, contrast-filled crater penetrating into the media, typically in older patients with extensive aortic disease, whereas ULP represents a focal intimal disruption within an IMH and may occur in patients without generalized atherosclerosis. PAU carries a higher risk of progression to dissection, aneurysm, or rupture, warranting closer surveillance/intervention.

Recent advances in imaging technology further refine the evaluation of AAS beyond conventional MDCT. Dual‑energy CT (DECT) enables the generation of virtual non‑contrast images, material decomposition, and iodine maps, which can reduce radiation exposure and improve characterization of IMH versus thrombus or contrast pooling.

Four-dimensional (4D) flow MRI is a new technique that offers time‑resolved, three‑dimensional visualization of blood flow patterns within the aorta and true/false lumens. This modality provides quantitative hemodynamic data, such as flow velocity, wall shear stress, and vortex formation, which may improve risk stratification, detect end-organ malperfusion, and guide treatment planning, especially in chronic or subacute presentations. It also carries the advantage of not exposing the patient to radiation.

Transesophageal echocardiography (TEE) can be used as the imaging modality in hemodynamically unstable patients or when CT angiography is not available. TEE provides high-resolution, real-time images of the aortic root, ascending aorta, and descending thoracic aorta, enabling accurate detection of proximal dissections (type A) and complications like aortic regurgitation or cardiac tamponade.

Current imaging recommendations from major bodies emphasize a multimodality approach tailored to clinical presentation and available resources. The American College of Radiology (ACR) appropriateness criteria (2022) consider CT angiography the first‑line imaging modality for suspected AAS due to its rapid acquisition, wide availability, and high diagnostic performance; MRI and TEE are appropriate alternatives in selected cases (when CT is contraindicated). The ACR criteria also support the use of ECG‑gated imaging to reduce motion artefacts in the thoracic aorta [13].

Recent guidelines from the European Society of Cardiology (ESC) in its peripheral arterial and aortic disease recommendations similarly stress early and accurate diagnosis with high‑resolution imaging, standardized measurement and reporting, and appropriate surveillance post‑intervention [14].

## Conclusions

AAS is a set of potentially fatal aortic conditions with a similar and overlapping clinical presentation. This case series illustrates the role of MDCT in the evaluation of AAS, highlighting its ability to provide high-resolution, multiplanar imaging of the aorta and its branches. MDCT allows disease classification, assesses disease extent, detects life-threatening complications such as rupture or organ malperfusion, and guides immediate management decisions, whether surgical, endovascular, or medical. It is important to note that due to resource limitations, this series primarily illustrates real-world imaging patterns of AAS in a rural hospital setting, rather than outcomes or management efficacy.
